# Automatic extraction channel of space debris based on wide-field surveillance system

**DOI:** 10.1038/s41526-022-00200-z

**Published:** 2022-05-05

**Authors:** Ping Jiang, Chengzhi Liu, Wenbo Yang, Zhe Kang, Cunbo Fan, Zhenwei Li

**Affiliations:** 1 Changchun Observatory of National Astronomical Observators, Chinese Academy of Sciences, Changchun, 130117 China; 2 University of Chinese Academy of Sciences, Beijing, 100049 China; 3 Key Laboratory of Space Object & Debris Observation, PMO, CAS, Nanjing, 210008 China

**Keywords:** Electrical and electronic engineering, Scientific data

## Abstract

In the past few years, the increasing amount of space debris has triggered the demand for distributed surveillance systems. Long exposure time can effectively improve the target detection capability of the wide-area surveillance system. Problems that also cause difficulties in space-target detection include large amounts of data, countless star points, and discontinuous or nonlinear targets. In response to these problems, this paper proposes a high-precision space-target detection and tracking pipeline that aims to automatically detect debris data in space. First, a guided filter is used to effectively remove the stars and noise, then Hough transform is used to detect space debris, and finally Kalman filter is applied to track the space debris target. All experimental images are from Jilin Observatory, and the telescope is in star-tracking mode. Our method is practical and effective. The results show that the proposed automatic extraction channel of space debris can accurately detect and track space targets in a complex background.

## Introduction

Since mankind launched the first satellite in 1957, a new era of space exploration has ushered in. However, more and more space activities have also brought a lot of space debris, including failed spaceflights as large as several meters in diameter, rocket wreckage, and projectiles in space missions, as small as a few centimeters and a few millimeters of solid rocket combustion and spacecraft in orbit collision disintegration of the debris^1,2^, and such pose serious threats to human space activities and the normal operation of satellites. Therefore, it is necessary to monitor the movement and orbit information of space debris to achieve effective prediction of its activities and avoid accidents. And space-target detection and tracking technology is the focus of our study^3,4^.

In many wide-field surveillance systems, detecting and tracking dim space targets from optical images has always been a problem. Owing to the long distance between the space target and the CMOS sensor, the target on the focal plane is characterized by weak intensity and small area and is often easily submerged by background and noise^5,6^. Therefore, many methods have been proposed to solve the problem of dim-target detection in images, such as the template matching method^7,8^, morphological algorithm^9,10^ and neural network^11^. Reed^12^ proposed 3D matched filtering to detect small moving targets with strong background clutter. This method has an excellent detection effect when the target has the same moving speed. However, the detection performance of targets with unknown speeds decreases sharply. Bai^13^ et al. used mathematical morphological operators to eliminate noise and background changes and finally merged them into a median image to obtain the position of the object. Blostein^14,15^ et al proposed a multistage hypothesis testing method, this algorithm first introduced a tree structure to represent the trajectory of the target, which can detect multiple targets at the same time. In order to reduce missed alarms, the establishment of multiple candidate trajectory starting points may lead to an exponential increase in subsequent branches, which seriously affects the performance of the algorithm.

## Methods

### Image-processing channel

**Fig. 1:**
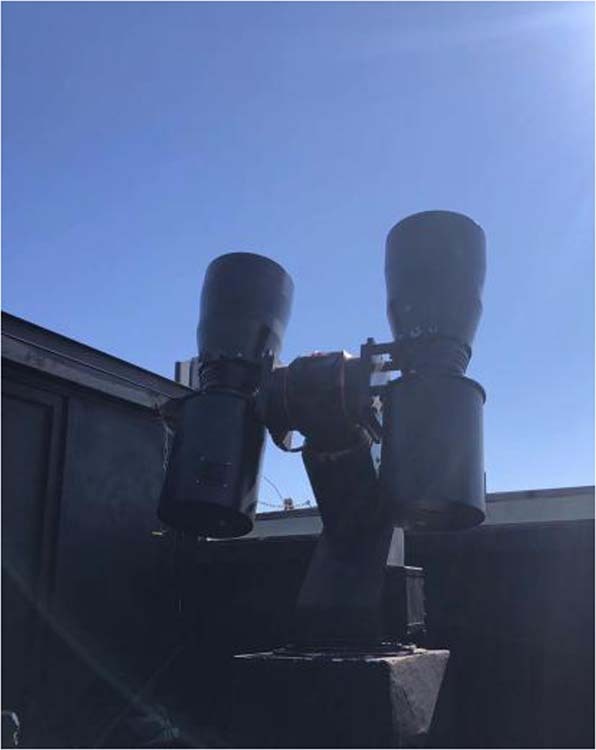
Space probe telescope components. This image was obtained by Changchun Observatory.

**Fig. 2:**
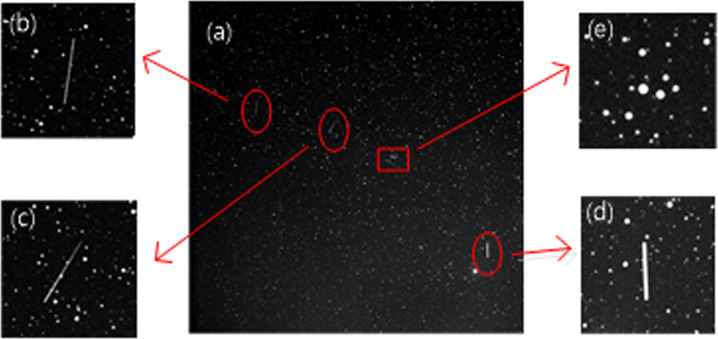
Magnifying regions of the target and star. **b**, **c** and **d** is the target of space debris. **e** is the star point. **a** Original image. **b**–**e** Truncated enlarged parts.

The astronomical image data used in the paper was collected by CMOS telescope. The specific parameters of the telescope are listed in Table 1. The time interval between exposures of the CMOS telescope camera is relatively short, and therefore it can be observed at a high frame rate. This camera has a relatively large field of view and a long detection range, and thus the captured astronomical images are relatively complex. These images were taken on the ground in star-tracking mode, in which the telescope was mounted on a turntable to counteract the speed of Earth’s rotation. Space objects appear as streaks, and stars appear as points in the image. Figure 1 shows the space exploration telescope components of the Changchun observatory. We have multiple telescopes that can cover a larger search area. Figure 2 shows the original astronomical image obtained by using this device.

**Table 1:** Parameters of the telescope.

Parameter	Value
Aperture of telescope	280 mm
Size of frame	4096 × 4096
Pixel size	9 × 9 µm
Field of view	6.5° × 6.5°
Weight	1.2 kg
Read Noise	3.7e−
Exposure time	2 s
Frame rate	0.5 Hz
Focal length	324 mm

The sequence image taken by the large field-based photoelectric telescope is a superposition of stars, space targets, and noise. It can be modeled as follows:

1 $$f(x,y,n) = O(x,y,n) + S(x,y,n) + B(x,y,n) + N(x,y,n)$$

where (*x*, *y*) represents the pixel coordinates in the star map. *n* represents the image frame number. *f*(*x*, *y*, *n*) represents the gray value of the image. *O*(*x*, *y*, *n*) represents the target gray value of the image. *S*(*x*, *y*, *n*) represents the star gray value of the image. *B*(*x*, *y*, *n*) represents the image background gray value. *N*(*x*, *y*, *n*) represents the noise gray value of the image, which may include atmospheric noise, space radiation, image generation noise, flicker noise, salt and pepper noise, multiplicative noise, and dark current^16,17^.

**Fig. 3:**
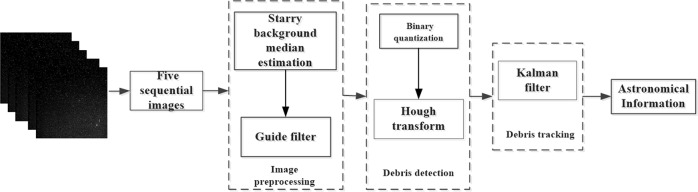
Structure diagram of space debris extraction channel. The flow chart completely describes the idea of the algorithm in the article.

Based on the above principles, we proposed schematic diagram of the debris information extraction algorithm is shown in Fig. 3. It is divided into three stages. The first step is to denoise the image and eliminate uneven background such as skylight. We obtain the median value of the multi-frame sequence star image to estimate the background intensity of the star image. Subsequently, we introduce guided filtering algorithms to further remove stars and isolated noise and then perform binary quantization. The second stage is space debris detection. First, binarization is applied to further process the image to reduce the amount of image data. Then apply Hough transform to detect space debris. The third part is space debris tracking. Kalman filter is applied for tracking to feedback debris information at all times.

### Image preprocessing

We first perform median filtering. The algorithm determines the gray level of the center pixel by sorting the pixels in the neighborhood. The median filter processing result will be largely affected by the set filter window size^18,19^. Small filter window can better protect the details of the original image, but the noise suppression capability will be limited. Large filter window can enhance the noise suppression capability, but the details of the original image will become blurred. We have improved the median filter algorithm to minimize the impact of the filter window size setting on the image processing results.

**Fig. 4:**
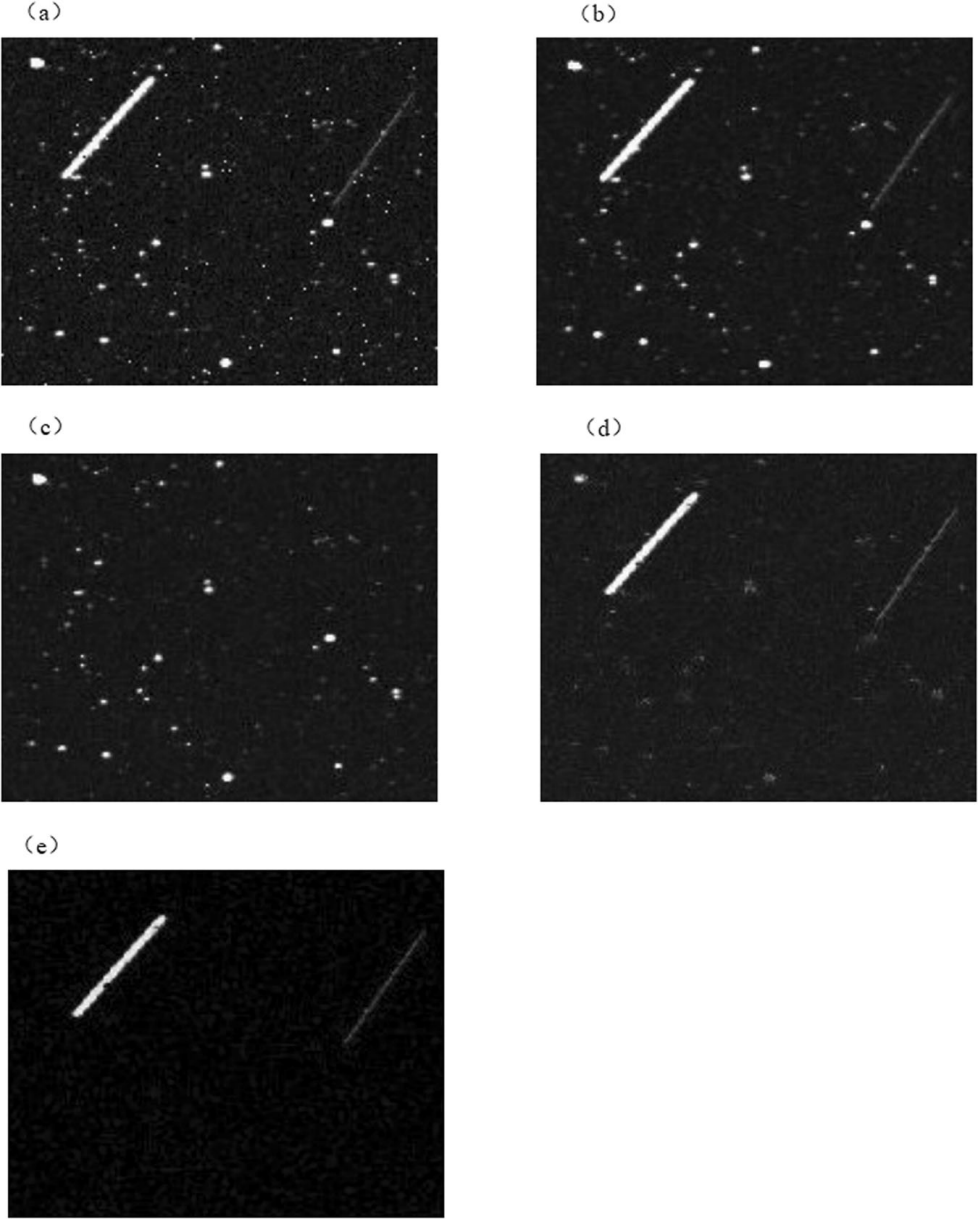
The experimental results of algorithm image processing. Image processing channels: **a** original image. **b** Median filtered image. **c** Background estimation image. **d** Background-subtracted image. **e** Guided filter denoising image.

First, 5 × 5 median filter is applied to process the original image, and the result of the processing is called the roughly denoised image. Calculate the average value of the rough noise-reduction image, the next step is to subtract the corresponding pixels of the original image from the average value, and the difference can be used as a threshold to judge the contaminated pixels in the image. The improved median filter only processes pixels that are considered to be contaminated by impulse noise by setting a threshold. Reducing the number of pixels processed by the filter window also reduces the influence of the filter window size setting on the image processing result. The contaminated pixels are processed with a median filter with size of 5 × 5, and the pixels that are not contaminated with noise remain unchanged, and the final improved median filter processing result is obtained. As shown in Fig. 4b, the median image was then analyzed for background estimation.

In order to detect the dim target in the image, we need to accurately estimate the uneven background. In astronomical images, we treat all pixels except the target as the background. The background analog-to-digital unit (ADU) value is the sum of photons from the sky plus the effects of various noises^20–22^. When processing the actual star map background, we take the median value of five consecutive star maps as the sky background. As shown in Fig. 4c, our algorithm accurately obtains most of the background, and the algorithm has the advantage of a small amount of calculation. We subtract the background image from the median filtered image to get an image containing space debris, isolated noise and stars, as shown in Fig. 4d, the image contains brighter stars and noise points, so in the next step we continue to eliminate these factors for the extraction target.

Guided filtering is an image smoothing filter based on a local linear model. The basic idea of the algorithm is to assume a linear relationship between each pixel in the image and its neighboring pixels, and obtain a linear model of each image, so as to obtain a basic image similar to the original image gradient^23^. In our extraction channel, the background suppressed image *g* is used as the input image, and *g* is also used as a guide image. When *g* is used as an input image and a guide image at the same time, the output of the guide filter can ensure the edge information of the space target. Its mathematical model is:

2 $$\begin{array}{*{20}{c}} {q_i = a_kg_i + b_k} & {\forall _i \in w_k} \end{array}$$

in the formula 2, *a*_*k*_ and *b*_*k*_ are the linear coefficients in window *w*_*k*_, *g* is the guide picture, *w*_*k*_ is the window with radius h, and the constraint equations of *a*_*k*_ and *b*_*k*_ in window *w*_*k*_ can be expressed as:

3 $$E(a_k,b_k) = \mathop {\sum}\limits_{i \in w_k} {((a_kg_i + b_k - p_i)^2 + \varepsilon a_k^2)}$$

in which *ε* is the regularization parameter, used to prevent the coefficient *a*_*k*_ from being too large. *p* represents the input picture. The coefficients *a*_*k*_ and *b*_*k*_ are respectively:

4 $$a_k = \frac{{\frac{1}{{\left| w \right|}}\mathop {\sum}\nolimits_{i \in w_k} {p_ig_i - \overline {p_k} u_k} }}{{\sigma _k^2 + \varepsilon }}$$

5 $$b_k = \overline {p_k} - a_ku_k$$

in the formula, *u*_*k*_ is the mean value in the window *w*_*k*_, $\sigma _k^2$ is the variance value in the window *w*_*k*_, $\overline {p_k}$ represents the mean value of the input image *p* in the window *w*_*k*_, and |*w*| represents the number of pixels in the window *w*_*k*_. As shown in Fig. 4e, we have obtained an image containing space debris and a very small number of noise points (which basically does not affect the extraction of debris).

**Fig. 5:**
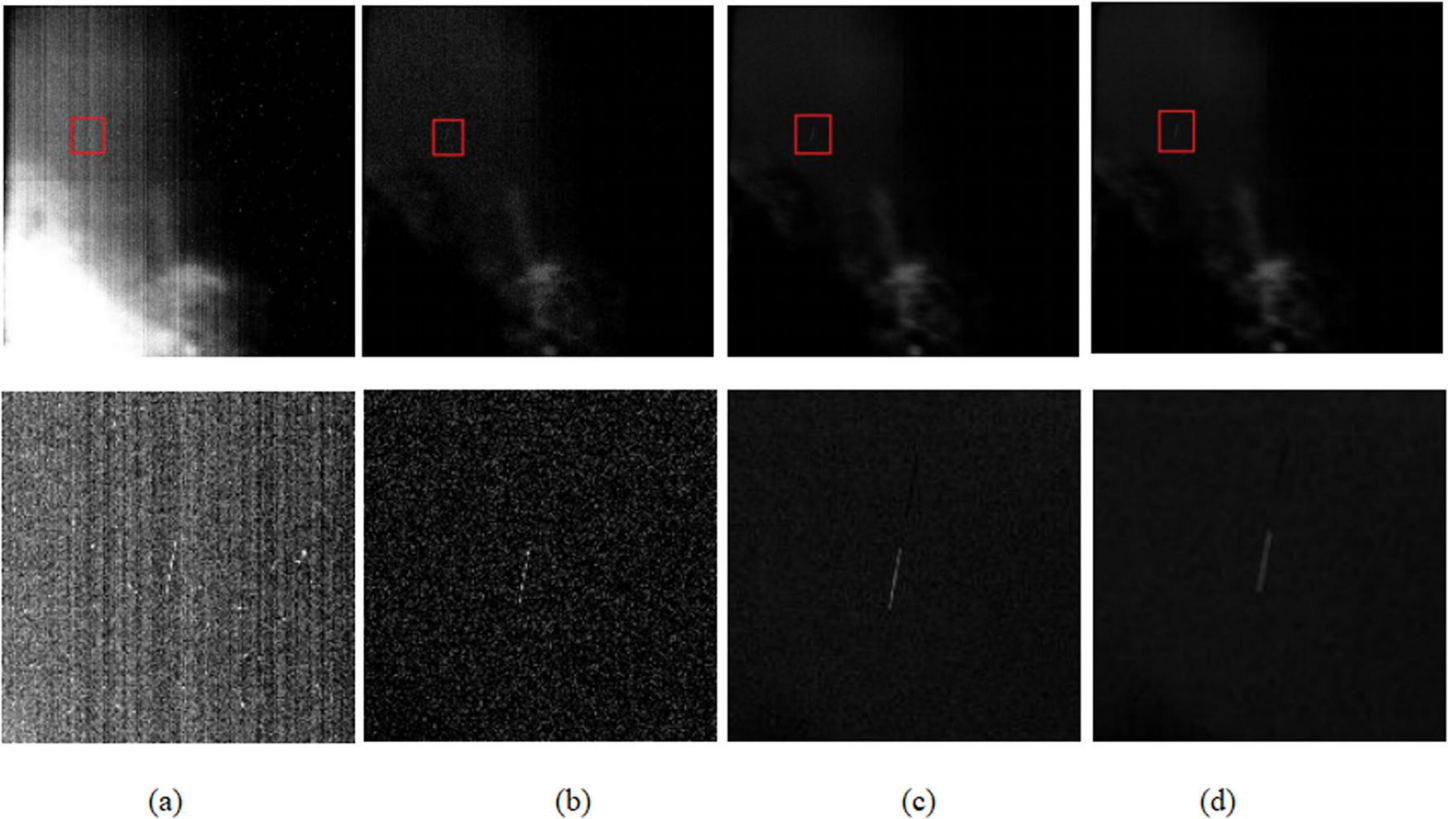
Top: the original image using different *h*-values for guided filter. Bottom: magnified part of the space debris target. In our implementation we set *h* = 0.3 H. Please zoom in to check details. **a** Original image. **b** *h* = 0.1 H. **c** *h* = 0.3 H. **d** *h* = 0.6 H.

It can be seen from the above formula that if the regularization parameter is set to a fixed value, the size of the filter window h will affect the quality of the star map. We select a star map with a large starlight background and noise to illustrate. Figure 5 shows the result of processing with multiple window sizes. When a smaller window is used (*h* = 0.1H, H represents the height of the image), there will be residual isolated noise and brighter stars, as shown in Fig. 5b. But h also cannot be too large, and the debris target (*h* = 0.6H) will be lost, as shown in Fig. 5d. In the experiment of this article, *h* = 0.3H is used, and the regularization parameter *ε* is set to 0.04, which has a good effect.

### Space debris detection

After the first stage of processing, we obtain candidate space targets through denoising algorithms and background suppression algorithms. We carry out the binarization process, which can reduces the amount of data, but also improve the precision of the Hough transform.

**Fig. 6:**
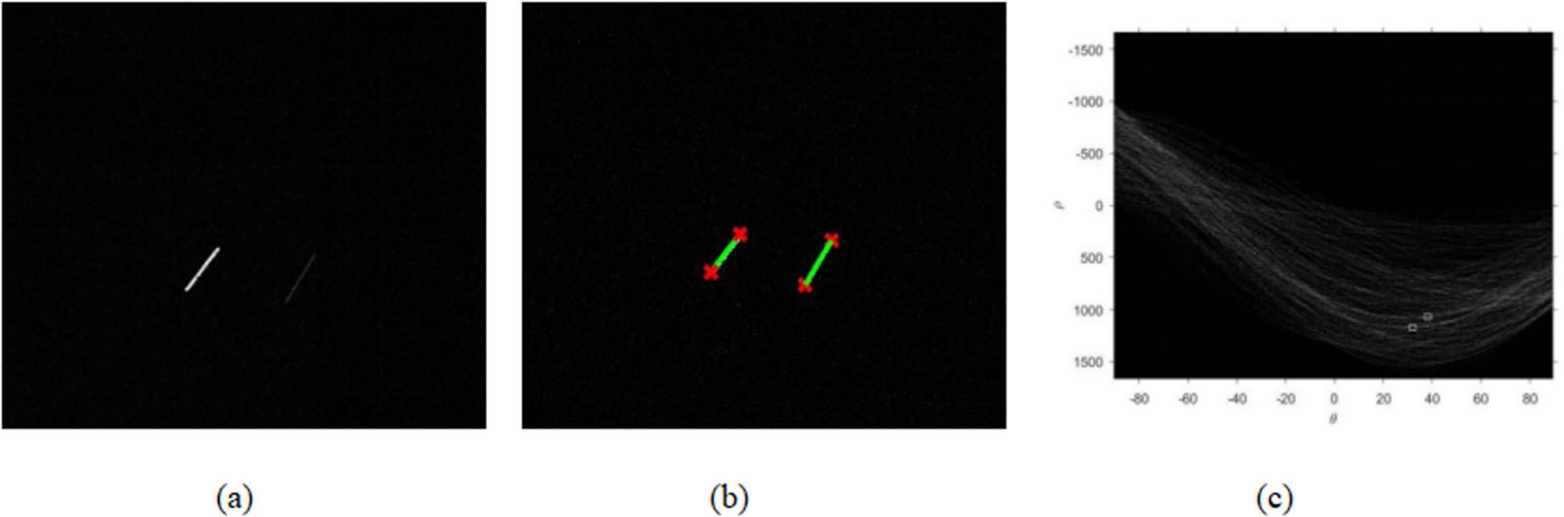
Hough transform detection result and parameter space curve. **a** Image before target detection. **b** Hough transform detection image **c** Parameter space map.

Binarization applies the maximum between-class variance method, which is the best algorithm for threshold selection. The threshold is the value with the largest variance between the target and background images. We use the Hough transform to process the image. This process converts the detection problem of a given curve in the original image into a problem of finding a peak in the parameter space. If fringes exist in the image, we map them to the parameter space and detect the peak value in it, as shown in the Fig. 6b. The Hough transform algorithm can detect the striped target in the image, so the starting point and ending point of the debris target can be obtained^24^.

When the line of common points in the image space is perpendicular to the x-axis, the slope is infinite, and the intersection point cannot be found in the Hough space. Therefore, the polar coordinates can be used. Similar to rectangular coordinates, the Hough transform in polar coordinates also transforms points in the image space into the parameter space. At this time, the obtained *ρ* and *θ* are the polar coordinate parameters of the straight line. For a straight line passing through any point (*x*, *y*) in the image space. Its polar coordinate space expression is:

6 $$\rho = x \ast \cos \theta + y \ast \sin \theta$$

in the formula 6, *ρ* is the vertical distance from the straight line to the origin, and *θ* represents the direction of the straight line. Once *ρ* and *θ* are determined, further analysis finds the endpoints and center of each line segment.

Figure 6c shows the curve distribution of the parameter space in the polar coordinate. The two space targets in the image have the most intersection points in the parameter space. Therefore, the points of collinear lines in the image space are mapped to the intersection points of the sine curves in the parameter space.

### Space debris tracking

When the space-debris target was detected in the first few frames of the images, we used the Kalman filter for follow-up tracking. Its small calculation amount and real-time calculation are more suitable for tracking space debris with a large field of view and a large amount of data. This method is an optimal linear recursive filtering method based on the minimum mean-square error, based on the state and the observation equations^25^. According to the movement characteristics of the debris in the large field of view optical observation system, it can be assumed that its movement approximately conforms to a uniform linear movement in adjacent frames. The initial position of the target is given by the detection result of the Hough transform, and the target speed can be given by the first two frames. The filter in the correction stage corrects the predicted value obtained in the prediction stage to obtain a new predicted value closer to the true value.

Prediction:

7 $$\begin{array}{l}{\mathop {x}\limits^{\Lambda ^\prime }} _n = A\,{\mathop {x}\limits^\Lambda} _{n - 1} + Bu_n\\ p_n^\prime = Ap_{n - 1}A^T + Q\end{array}$$

**Fig. 7:**
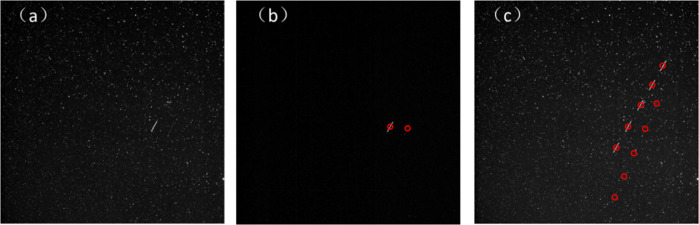
Tracking image and multi-frame trajectory image. **a** Original star map. **b** Kalman tracking image. **c** Kalman tracking trajectory diagram. The two objects in the figure are moving in opposite directions.

Correction:

8 $$\begin{array}{ll}k_n = p_n^\prime H^T(Hp_n^\prime H^T + R)^{ - 1}\\ {\mathop {x}\limits^\Lambda} _n = {\mathop {x}\limits^{\Lambda ^\prime }} _n + K_n{\mathop {Z}\limits^\Lambda} _n\\ p_n = (I - K_nH)P_n^\prime \end{array}$$

in which ${\mathop {x}\limits^\Lambda} _n$ and ${\mathop {x}\limits^\Lambda} _{n - 1}$ represent the true value, ${\mathop{x}\limits^{\Lambda\prime}}_n$ represents the Kalman estimated value; *p*_*n*_ is the Kalman error covariance matrix; $P_n^\prime$ is the Kalman estimation error covariance matrix; *k*_*n*_ is the Kalman gain matrix; $\mathop {Z}\limits^\Lambda _n$ is the measured value; *A* represents State transition matrix; *B* represents the input control matrix; *p* represents the error matrix; *Q* represents the predictive noise covariance matrix; *R* represents the observation noise covariance matrix; *H* represents the observation matrix. Figure 7b shows the Kalman tracking graph. It can be seen from the figure that the algorithm can track the detected debris targets well without losing the weaker targets. Figure 7c shows the algorithm tracking trajectory graph, and there is no target loss.

### Experiment

In this part, we will conduct some experiments to verify the performance of the algorithm. The images used in the experiments are all obtained by the large field of view telescope in the star-tracking mode. The background stars in the image are dense, and it is necessary to accurately extract the streak target, and the image size is 4096 × 4096 pixels. For ease of understanding and clarity, some of the experimental images have been reduced. In this summary, the pseudocode of debris extraction channel and the detection effect of real astronomical images are illustrated.

### Procedure

As shown in Table 2, in this section we give the pseudo-program of the detection algorithm.

**Table 2:** The pseudocode of the our detection method is channel 1.

Channel 1. Automated space debris extraction channel
Input: Raw star map sequence I.
1. Raw star map sequence.
Parameter: Improved median filter window is set to 5×5 window size. We set the guide filtering window is 0.3 h.
Initialization: After median filtering is H, Multi-frame median processing g, The guided filter eliminates star points is K
2. Space debris detection and tracking
Parameter: The output of the Hough transform is ${\mathop {x}\limits^ \wedge} _{n - 1}$, Kalman estimate${\mathop {x}\limits^{ \wedge \prime }} _n$, Estimate error covariance matrix $P_n^\prime$, Error covariance matrix $P_n$.
Initialization:$$p_{n - 1} \to p_n^\prime$$_,_ $$p_n^\prime \to k_n$$_,_ $${\mathop {Z}\limits^ \wedge} _n \to {\mathop {x}\limits^ \wedge} _n$$
With ${\mathop {x}\limits^ \wedge} _{n - 1}$ and $P_{n - 1}$,solve for $\mathop {x}\limits^ \wedge _n$ and $P_n^\prime$ in Eq. 7
With H and R, solve for $K_n$ with Eq. 8
Return $${\mathop {x}\limits^{ \wedge \prime }} _n$$ and $$P_n$$
Output: Real-time target tracking and location information

### Real astronomical image detection experiment

**Fig. 8:**
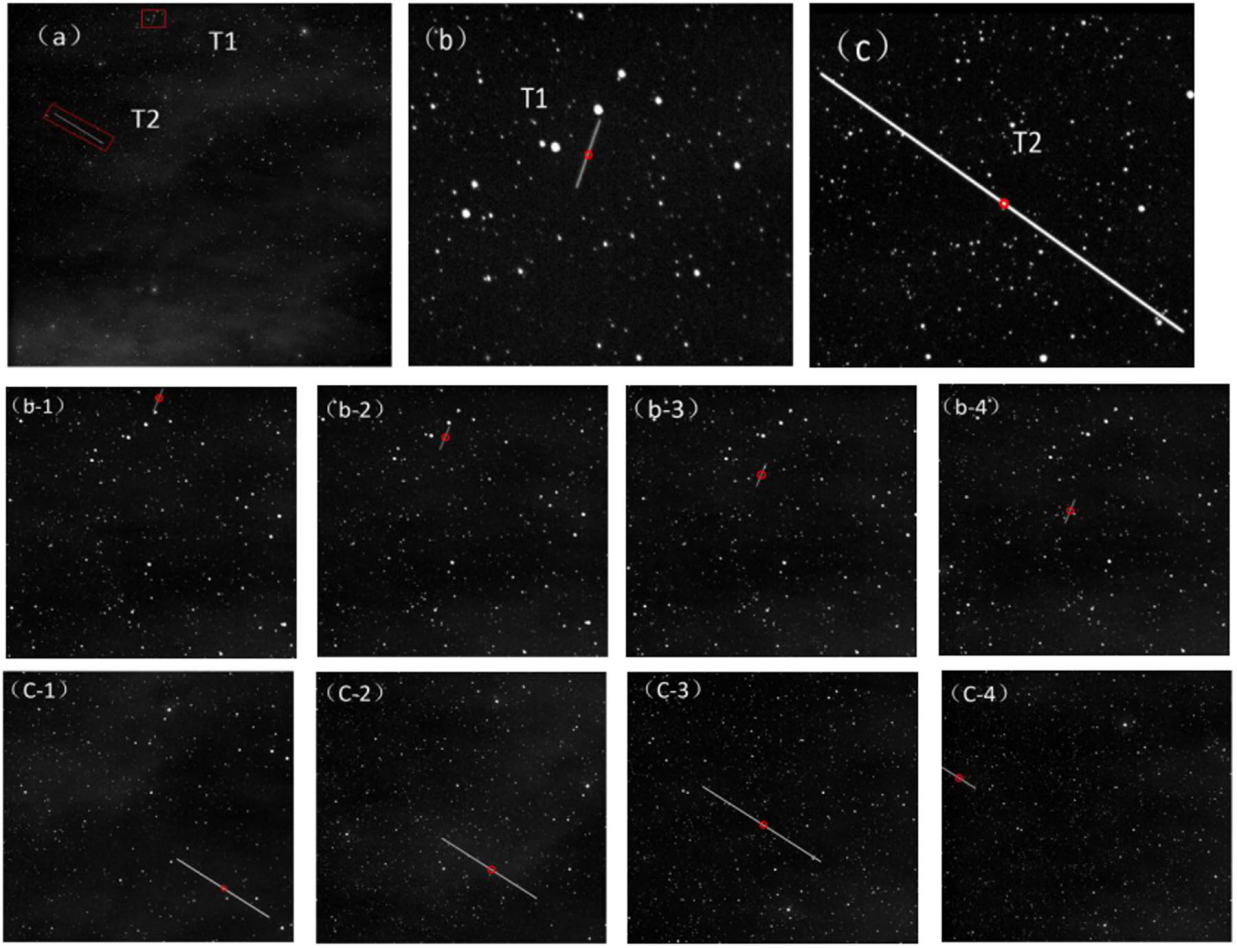
Experimental effect image. (frames 1–4). **a** The first frame of star map target detection results. **b** and **c** are the T1 and T2 targets in the red box clearly displayed. **b-1**–**b-4** and **c-1**–**c-4** display the continuous frame image tracking results of the target T1 and T2.

The real astronomical image used in this experiment to explain the detection and tracking effect of the method. In order to fully test the algorithm proposed in this paper, the targets are named T1 and T2 as shown in the figure, and the space target detection results are shown. The red circles display the detected targets. Figure 8 shows the experimental results, b-1 to b-4 are the detection and tracking of the target T1, and c-1 to c-4 display the experimental result of the target T2.

**Fig. 9:**
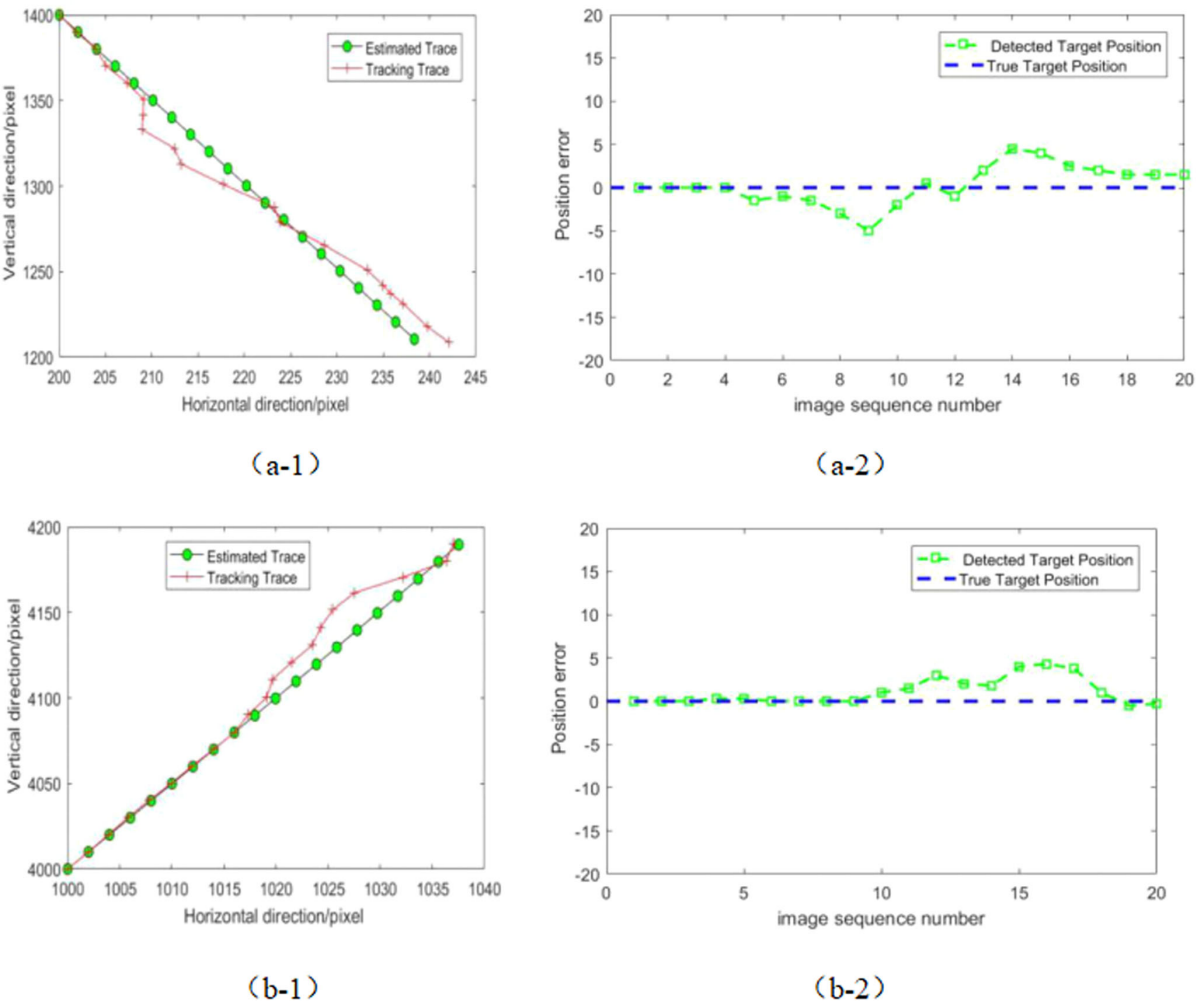
Debris target trajectory tracking curves of experimental statistics. **a-1** represents the multi-frame true trajectory of the T1 target and the trajectory curve detected by the algorithm. **a-2** represents the target multi-frame centroid position error of T1. **b-1** represents the multi-frame true trajectory of the T2 target and the trajectory curve detected by the algorithm. **b-2** represents the target multi-frame centroid position error of T2.

It can be seen from the detection results that the algorithm detects the target in four consecutive frames of images, the algorithm can continuously track target with high precision, which shows the practicability and stability of the algorithm. Figure 9 shows the estimation and tracking of the centroid coordinates of continuous multi-frame images of our algorithm. We can see that the algorithm has a better tracking effect. This shows the excellent detection performance and real-time tracking capability of the debris extraction channel.

To further demonstrate the performance of the proposed space debris extraction approach, we apply the multistage hypothesis testing algorithm (MHT)^14^, new top-hat algorithm (NTH)^26^, improved maximum value projection algorithm (IMVP)^27^ and our extraction channel to extract space debris from the same actual star image, and analyze the probability of debris extraction, false alarm rate and time-consuming process. The evaluation results of the images are given in Table 3. In order to compare the fairness of the results, the selected comparison methods are all carried out using standard published parameters. In the MHT method, detection is performed on all space target trajectories in the star map. However, with the increase of noise and the number of targets, the trajectory of targets increases sharply, resulting in a large amount of computational cost. In the wide-area monitoring system, the NTH detection result is affected by structural elements, which seriously affects the performance of the algorithm. The IMVP algorithm can detect moving targets well and track their trajectories accurately, but the algorithm comes at the cost of computation.

**Table 3:** Statistical results of real space target detection.

Method	Detection probability	False alarm rate	Running time
MHT	87.7%	17.2%	36.94 s
NTH	90.6%	69.1%	6.8 s
IMVP	91.4%	15.9%	10.54 s
OURS	96.2%	8.4%	4.32 s

**Fig. 10:**
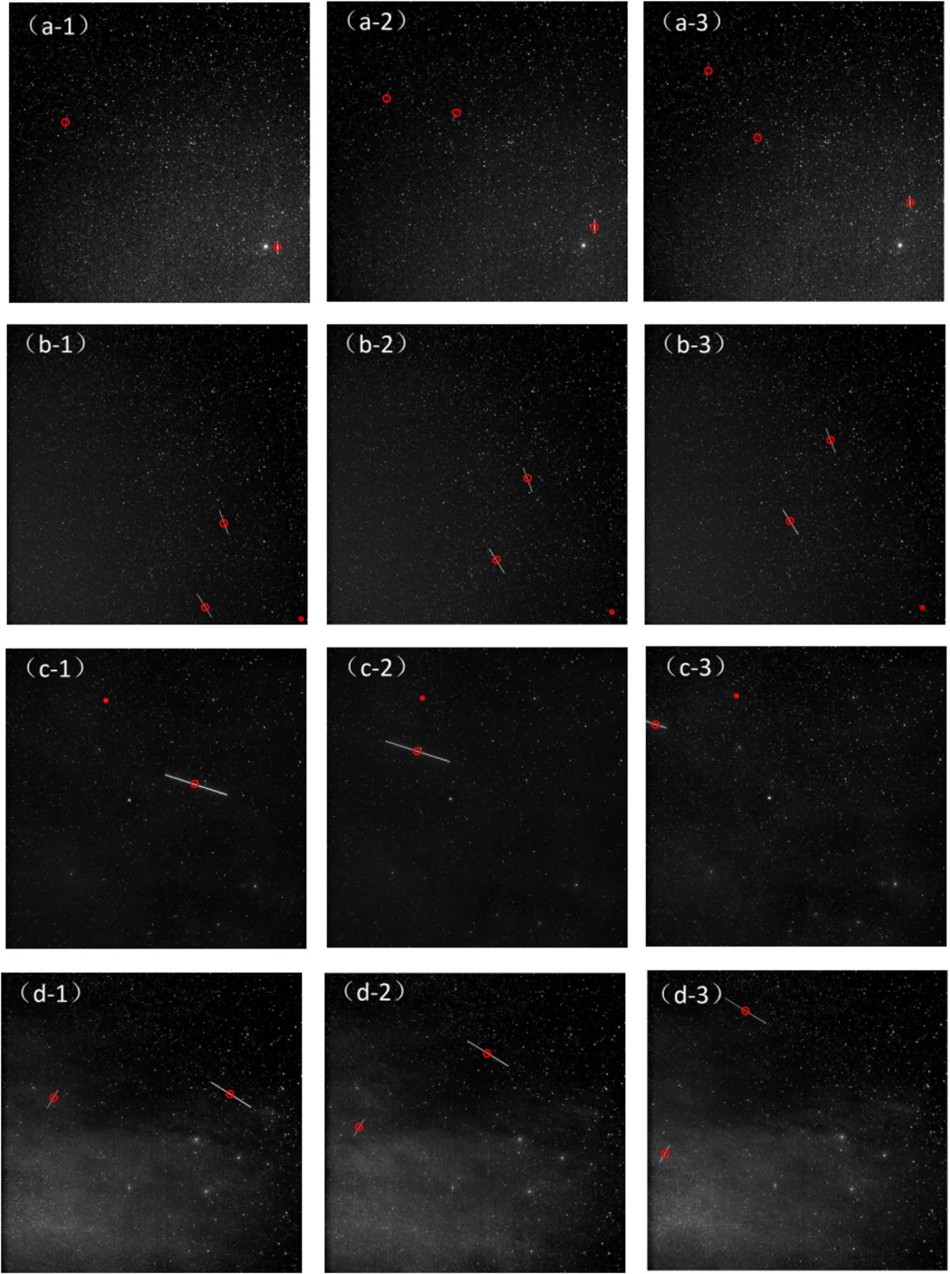
Detection results of different astronomical images. We selected four types of astronomical images with different backgrounds for debris detection and tracking.

**Fig. 11:**
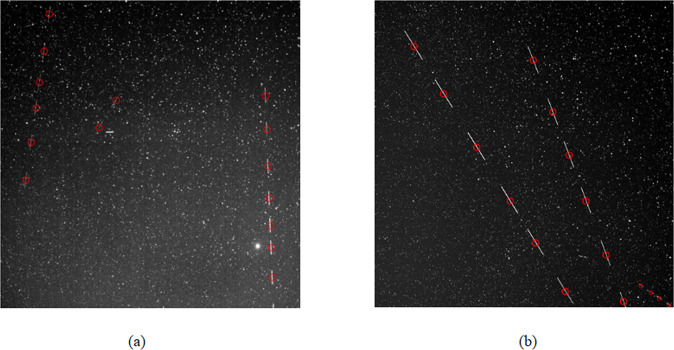
The experimental research on actual astronomical image data. **a** represents the image target trajectory of a-1 in Fig. 10. **b** represents the continuous multi-frame image target trajectory of b-1 in Fig. 10. **a-1**–**a-3** represent the detection and tracking results of the algorithm at three consecutive moments. The same rule applies to **b-1**–**b-3**, **c-1**–**c-3** and **d-1**–**d-3**.

Our algorithm has low false alarm rate and simple calculation. The channel was evaluated for 200 real images. Figure 10 shows the detection results of four images with different backgrounds. The images from a-1 to a-3 and b-1 to b-3 have more stars and noise, the images from c-1 to c-3 have less noise, and the images from d-1 to d-3 are not only composed of a large number of the stars and noise are also disturbed by clouds. Our algorithm has obtained better detection and tracking effects in these types of images. Figure 11 shows the debris trajectory diagrams of Fig. 10a, b. The target trajectory is circled in red. The proposed debris extraction channel can extract space debris very well and takes less time.

## Results

The space target information automatic extraction pipeline is research in this paper. The algorithm is suitable for the detection of space stripe targets with complex background. The median value of multiple frames of images is taken to suppress the influence of the background, and guided filtering is introduced to eliminate isolated noise points and stars. The Hough transform and Kalman filter were used for target detection and tracking. We conducted tests on the original star map. The test results show that our space debris extraction channel can not only effectively detect debris targets, but also accurately track debris information.

## Discussion

In our approach, the multi-frame median method is used to suppress the influence of the background, and guided filtering is introduced to eliminate isolated noise points and stars. Hough transform is used to detect space debris, and finally Kalman filter is applied to track the space debris target. We propose a channel to detect the presence of space debris without the need to use auxiliary space targets and orbital data. We did not obtain information in advance to help the target detection algorithm. Furthermore, the focus of our work is not only the debris detection technology itself, but the space target information extraction that combines space debris detection and astronomical positioning.

### Reporting summary

Further information on research design is available in the Nature Research Reporting Summary linked to this article.

## Supplementary information

Reporting Summary

## Data Availability

All image data in the article are raw data, not obtained from public databases, if necessary, all data in the article can be obtained from the corresponding author upon reasonable request.
