# Supplementary material for: Automatic extraction channel of space debris based on wide-field surveillance system
Source: NPJ Microgravity. 2022 May 5;8:14. doi: 10.1038/s41526-022-00200-z (PMC9072332; doi:10.1038/s41526-022-00200-z)
Supplement: Supplementary file 1 — Reporting Summary [file 41526_2022_200_MOESM1_ESM.pdf]

nature portfolio

Corresponding author(s): Chengzhi Liu

Last updated by author(s): 21/01/2022

Reporting Summary

Nature Portfolio wishes to improve the reproducibility of the work that we publish. This form provides structure for consistency and transparency in reporting. For further information on Nature Portfolio policies, see our Editorial Policies and the Editorial Policy Checklist.

Statistics

For all statistical analyses, confirm that the following items are present in the figure legend, table legend, main text, or Methods section.

n/a	Confirmed	
☐	☒	The exact sample size (n) for each experimental group/condition, given as a discrete number and unit of measurement
☐	☒	A statement on whether measurements were taken from distinct samples or whether the same sample was measured repeatedly
☐	☒	The statistical test(s) used AND whether they are one- or two-sided<br>*Only common tests should be described solely by name; describe more complex techniques in the Methods section.*
☐	☒	A description of all covariates tested
☐	☒	A description of any assumptions or corrections, such as tests of normality and adjustment for multiple comparisons
☐	☒	A full description of the statistical parameters including central tendency (e.g. means) or other basic estimates (e.g. regression coefficient) AND variation (e.g. standard deviation) or associated estimates of uncertainty (e.g. confidence intervals)
☐	☒	For null hypothesis testing, the test statistic (e.g. F, t, r) with confidence intervals, effect sizes, degrees of freedom and P value noted<br>*Give P values as exact values whenever suitable.*
☐	☒	For Bayesian analysis, information on the choice of priors and Markov chain Monte Carlo settings
☐	☒	For hierarchical and complex designs, identification of the appropriate level for tests and full reporting of outcomes
☐	☒	Estimates of effect sizes (e.g. Cohen's d, Pearson's r), indicating how they were calculated

Our web collection on statistics for biologists contains articles on many of the points above.

Software and code

Policy information about availability of computer code

Data collection	The code and data used in the paper are written by ourselves, and no commercial or public code is involved.
Data analysis	The code and data used in the paper are written by ourselves, and no commercial or public code is involved.

For manuscripts utilizing custom algorithms or software that are central to the research but not yet described in published literature, software must be made available to editors and reviewers. We strongly encourage code deposition in a community repository (e.g. GitHub). See the Nature Portfolio guidelines for submitting code & software for further information.

Data

Policy information about availability of data

All manuscripts must include a data availability statement. This statement should provide the following information, where applicable:
- Accession codes, unique identifiers, or web links for publicly available datasets
- A description of any restrictions on data availability
- For clinical datasets or third party data, please ensure that the statement adheres to our policy

All data in the article can be obtained from the corresponding author under reasonable request.

Field-specific reporting

Please select the one below that is the best fit for your research. If you are not sure, read the appropriate sections before making your selection.

☐ Life sciences ☐ Behavioural & social sciences ☒ Ecological, evolutionary & environmental sciences

For a reference copy of the document with all sections, see nature.com/documents/nr-reporting-summary-flat.pdf

Ecological, evolutionary & environmental sciences study design

All studies must disclose on these points even when the disclosure is negative.

Study description	Collect data through actual astronomical observatories, and propose a set of space debris extraction methods that meet the requirements to avoid the interference of space debris
Research sample	The Changchunwen Observatory has a number of advanced astronomical observation equipment, and the observatory is dedicated to the extraction of space debris and has profound experience.
Sampling strategy	When selecting data, multiple classic dim fragments are used for extraction, and multiple sets of comparative experiments show that the algorithm has excellent performance.
Data collection	The data is collected and sorted by Dr. Ping jiang
Timing and spatial scale	Data collection On May 24, 2021, we collected the data overnight through the optoelectronic equipment of the observatory, and sorted it out
Data exclusions	We have conducted a lot of experimental data analysis, in order to fully illustrate the applicability of the algorithm
Reproducibility	All repeated experiments are successful
Randomization	All data have been carried out at least three experiments, and all experimenters participated in the whole process
Blinding	All experiments have been accurately analyzed and calculated

Did the study involve field work? ☐ Yes ☒ No

Reporting for specific materials, systems and methods

We require information from authors about some types of materials, experimental systems and methods used in many studies. Here, indicate whether each material, system or method listed is relevant to your study. If you are not sure if a list item applies to your research, read the appropriate section before selecting a response.

Materials & experimental systems

n/a	Involved in the study
☒	☐ Antibodies
☒	☐ Eukaryotic cell lines
☒	☐ Palaeontology and archaeology
☒	☐ Animals and other organisms
☒	☐ Human research participants
☒	☐ Clinical data
☒	☐ Dual use research of concern

Methods

n/a	Involved in the study
☒	☐ ChIP-seq
☒	☐ Flow cytometry
☒	☐ MRI-based neuroimaging
